# Protective Effect of Shen-Fu Injection on Neuronal Mitochondrial Function in a Porcine Model of Prolonged Cardiac Arrest

**DOI:** 10.1155/2014/523847

**Published:** 2014-11-19

**Authors:** Wei Gu, XiaoMin Hou, Haijiang Zhou, ChunSheng Li

**Affiliations:** Department of Emergency Medicine, Beijing Chaoyang Hospital, Capital Medical University, No. 8 Worker's Stadium South Road, Chaoyang District, Beijing 100020, China

## Abstract

*Background*. Shen-Fu injection (SFI) following cardiac arrest exhibits neurological effects, but its effect on neurological dysfunction is unclear. This study sought to investigate the protective effect of SFI on nerve cells in a porcine model of cardiac arrest. *Methods*. After eight minutes of untreated ventricular fibrillation (VF) and 2 minutes of basic life support, 24 pigs were randomized and divided into three cardiopulmonary resuscitation groups, which received central venous injection of either Shen-Fu (SFI group; 1.0 ml/kg), epinephrine (EP group; 0.02 mg/kg), or saline (SA group). Surviving pigs were sacrificed at 24 h after ROSC and brains were removed for analysis for morphologic changes of mitochondria by electron microscopy, for mitochondrial transmembrane potential (MTP) by flow cytometry, and for opening of the mitochondrial permeability transition pore (MPTP) by mitochondrial light scattering. *Results*. Compared with the EP and SA groups, SFI treatment reduced opening of MPTP, showing higher MMP. In addition, animals treated with SFI showed slight cerebral ultrastructure damage under the electron microscopy. *Conclusion*. Shen-Fu injection alleviated brain injury, improved neurological ultrastructure, stabilized membrane potential, and inhibited opening of MPTP. Therefore, SFI could significantly attenuate postresuscitation cerebral ischemia and reperfusion injury by modulating mitochondrial dysfunction of nerve cells.

## 1. Introduction

Less than 10% of patients admitted to hospital after successfully resuscitated out-of-hospital cardiac arrest (OHCA) will leave hospital without major neurological impairments [1]. Two-thirds of patients after successful cardiopulmonary resuscitation (CPR), even after active treatment, will have difficulty in avoiding neurological sequelae, including postanoxic vegetative state and delayed death [2]. Although mild hypothermia is the only effective treatment, clinical and animal experiments confirmed that it improves neurological outcome in patients with cardiac arrest coma [3]. To date, a growing body of research focuses on neuroprotective treatment after postresuscitation cerebral injury and there is clinical evidence that early drug treatment can improve neurological function and survival after discharge from CPR [4].

Cardiac arrest (CA) results in whole-body ischemia-reperfusion injury and represents the most severe shock state, during which the delivery of oxygen is abruptly halted. The nervous system is sensitive to hypoxia ischemia. Complete cerebral hypoxia occurred 5 min after CA. Energy of neurons will be exhausted and metabolites accumulate in the cell and the imbalance of cell membrane pump function occurs. Then the cell homeostasis is severely damaged, which subsequently can cause brain cell necrosis. Although resuscitation requires reperfusion of ischemic tissue with oxygenated blood to restore aerobic metabolism and organ function, reperfusion concomitantly activates multiple pathogenic mechanisms, collectively known as “reperfusion injury.” At the center of reperfusion injury are mitochondria, playing a critical role as effectors and targets of injury [5]. Mitochondrial dysfunction is considered to be key determinant with respect to the extent of injury during cerebral ischemia. Impairment of mitochondrial function leads to reduced ATP production, impaired calcium buffering, and overproduced reactive oxygen species [6].

Shen-Fu injection (SFI) is a well-known traditional Chinese herbal medicine containing* Ginseng *and* Aconitum *extracts and has been commonly used in China for nearly 800 years [7]. Shen-Fu injection is a typical form of SFI decoction for intravenous medication, whose main components include ginsenoside (0.8 mg/mL) and aconitine (0.1 mg/mL) [7]. Its quality is controlled strictly according to the standard of China Ministry of Public Health, and fingerprint technology was adopted in the process of production to ensure its quality. The effects of SFI are based on aconitine properties, supplemented by ginsenoside, which can increase heart rate and myocardial contractility and promote a reduction in blood pressure. Aconite contains noradrenaline salsolinol, which has excitatory effects on p receptors and *α*-adrenergic receptors, which can significantly increase cerebral blood flow by improving mean arterial pressure (MAP) [8]. SFI has been clinically used for treatment of many kinds of diseases, including coronary artery dilation, shock, and improved heart function [9, 10]. Previous studies proved that SFI had protective effect on postresuscitation lung and myocardial injury by modulating apoptosis [7, 11]. Our previous experiments demonstrated that SFI reduced cerebral damage in a porcine model of CA, which may be related to suppression of the inflammatory reaction and decrease of brain edema after ROSC. Furthermore, SFI was reported to be neuroprotective after cerebral ischemia in the rat [12].

However, the mechanisms responsible for the neuroprotective effects of SFI are not well understood. Therefore, in the present study, we employed a swine model of cardiac arrest to explore the postresuscitation mitochondrial mechanism and the protection effects of SFI on swine nerve cells.

## 2. Methods

### 2.1. Animal Preparation

#### 2.1.1. Experimental Animal

Thirty inbred Wuzhishan miniature pigs (12–14 months of age, 30 ± 2 Kg), purchased from Chinese Academy of Agricultural Sciences (license number: SYXK (Beijing) 2008-0007), were used in this study. The use of experimental animals complied with laboratory animal use regulations of Capital Medical University and the study was conducted after receiving the approval from the Animal Care Committee of Capital Medical University (license number: 2010-D-013).

#### 2.1.2. Animal Grouping

Thirty pigs were randomized into four groups: (1) Shen-Fu injection group (SFI, *n* = 8): CA 8 min, central venous injection of Shen-Fu injection (1.0 mL/Kg); (2) epinephrine group (EP, *n* = 8): CA 8 min, central venous injection of epinephrine (0.02 mg/Kg); (3) saline group (SA, *n* = 8): CA 8 min, central venous injection of saline (SA); (4) Sham operation group (SHAM, *n* = 6). The same procedure without CA initiation was achieved in the sham operation group, including induction of anesthesia, electrode positioning, mechanical respiration, and monitoring of physiological parameters.

### 2.2. Reparation and Intervention of Animal Model

#### 2.2.1. Preoperative Preparation

The animals were fasted overnight but had free access to water. Anesthesia was induced by intramuscular injection of midazolam ketamine (0.5 mg/Kg), followed by an injection of propofol (1.0 mg/Kg) into the ear vein and an injection of pentobarbital (8 mg/Kg/h) to maintain anesthesia. A cuffed 6.5 mm endotracheal tube was advanced into the trachea and animals were mechanically ventilated in a volume-controlled ventilator (Servo 900c; Siemens, Berlin, Germany), using a tidal volume of 10 mL/Kg and a respiratory frequency of 12/min on room air. End-tidal PCO_2_ was monitored with in-line infrared capnography (CO2SMO plus respiratory monitor; Respironics Inc., Murrysville, PA, USA). The respiratory frequency was adjusted to maintain end-tidal PCO_2_ between 35 and 40 mmHg before VF was induced.

#### 2.2.2. Arteriovenous Catheterization

After the left femoral artery was isolated by layer, a Swan-Ganz catheter (7F; Edwards Life Science, Irvine, CA) was advanced from the left femoral vein and flow-directed into the pulmonary artery to measure cardiac output (CO). MAP was measured with a fluid-filled catheter that was advanced from the left femoral artery into the thoracic aorta. A 6F pressure catheter was inserted into the right femoral artery to measure MAP. A 5F pacing catheter was advanced from the right internal jugular vein into the right ventricle to induce ventricular fibrillation (VF). The electrocardiogram and all hemodynamic parameters were monitored with a patient monitoring system (M1165; Hewlett-Packard, Palo Alto, CA).

#### 2.2.3. Induction of Ventricular Fibrillation

After surgery, the animals were allowed to equilibrate for 30 minutes to achieve a stable resting level. The temporary pacemaker conductor was inserted into the right ventricle through the right sheathing canal and connected to an electrical stimulator (GY-600A; Kaifeng Huanan Equipment Co., Ltd., China) programmed in the S1S2 mode (300/200 ms), 40 V, 8 : 1 proportion, and 10 ms step length to provide a continuous electrical stimulus until VF. VF was defined as an electrocardiogram showing waveforms corresponding to VF and a rapid decline in mean aortic pressure (MAP) toward zero. Ventilation was stopped while inducing VF [8].

#### 2.2.4. Cardiopulmonary Resuscitation

After 8 minutes of VF, manual CPR was carried out at a frequency of 100 compressions per minute with mechanical ventilation at a FiO_2_ of 100% and a compression-to-ventilation ratio of 30 : 2. The quality of chest compressions was controlled by a HeartStart MRx Monitor/Defibrillator with Q-CPR (Philips Medical Systems, Best, Holland). After 2 minutes of CPR, pigs were randomly divided into 3 groups and then received central venous injection of Shen-Fu injection (1.0 mL/Kg), epinephrine (0.02 mg/Kg), and saline, respectively. If the spontaneous circulation was not restored, defibrillation was performed once with a diphase 150 J. If spontaneous circulation was still not achieved, CPR was continued for a further 2 minutes and defibrillation was performed once more until ROSC. ROSC was defined as 10 consecutive minutes of maintenance of systolic blood pressure at 50 mmHg [13]. If spontaneous circulation was not restored within 30 minutes, we regarded the animal as dead.

#### 2.2.5. Treatment before and after ROSC

Pigs were infused saline (10 mL/Kg*·*h) during operation and supplied by saline infusion, 5% glucose, and sodium chloride injection after operation according to postoperative central venous pressure (CVP) and urine volume, the required liquid, in addition, with the physiological requirements of potassium chloride 3–5 g, the CVP was maintained at 5–12 mmHg. At 6 hours after ROSC, without any anesthetic and sedative drugs, ventilator was removed from the pigs and the pigs were placed into the feeding cage. Room temperature was maintained at between 20 and 24°C in the process of the experiment.

The animals were euthanized with 10 mL of 10 mol/L potassium chloride intravenously following a bolus of 100 mg of propofol intravenously at 24 h after resuscitation.

### 2.3. Measurements

Hemodynamic parameters including heart rate, CO, and MAP were measured continuously. We recorded the values at baseline; 30 min; and 1, 2, 4, and 6 h after ROSC.

Some brain specimens were removed for analysis for ultrastructure by transmission electron microscopy, mitochondrial transmembrane potential (MTP) by flow cytometry, and opening of the mitochondrial permeability transition pore (MPTP) by mitochondrial light scattering.

### 2.4. Neurological Deficit Scores (NDS) and Cerebral Performance Categories (CPC) Scores

All porcine CPC and NDS were used to evaluate preliminary neurological function at 24 h after recovery. The CPC evaluation consists of a 5-point scale to assess neurological function [14]. NDS including the level of consciousness, breathing pattern, cranial nerve function, and sensory and motor function.

### 2.5. Detection of Brain Mitochondrial Function

#### 2.5.1. Isolation of Mitochondria

Mitochondria were isolated from pig brain tissues. Brain tissues were minced on ice in 5 mL of ice-cold isolation medium and then manually homogenized using a glass homogenizer. Thereafter, the homogenate was added to 15 mL of ice-cold isolation medium and centrifuged at 1500 ×g at 4°C for 4 minutes. After centrifugation, the supernatant was filtered using cheesecloth and centrifuged again at 10,000 ×g at 4°C for 10 mins. Mitochondrial protein was determined using the BCA method.

#### 2.5.2. Detection of Mitochondrial Membrane Potential (MMP)

The 90 *μ*L GENMED staining solution containing staining solution (Reagent B) and dilution (Reagent C) was placed into each hole of 96-hole plate; then 10 *μ*L purified mitochondria sample was added, gently shaked, and placed in dark room. The mitochondria were then left to incubate for 10 minutes at room temperature in the dark. After incubation, the fluorescence of the sample was immediately analyzed with a spectrofluorimeter (Infinite M200; Tecan) at an excitation wavelength of 490 nm and emission wavelength of 590 nm.

#### 2.5.3. Determination of Mitochondrial Permeability Transition Pore (MPTP)

Mitochondria (0.5 mg protein/mL) were suspended in 2 mL incubation buffer (125 mmol/L KCl, 2 mmol/L K_2_HPO_4_, 1 mmol/L MgCl_2_, 1 *μ*mol/L EGTA, 20 mmol/L Tris, pH 7.2, 5 mmol/L malic acid, 5 mmol/L glutamate) at 37°C in a water jacketed cuvette holder. Mitochondrial swelling was triggered by the addition of CaCl_2_ (10 *μ*mol/L). After a 1-minute equilibration period, mitochondrial swelling was assessed in a spectrophotometer via the decrease in absorbance at 540 nm. Measurements were repeated every 30 seconds for 10 minutes.

### 2.6. Statistical Analysis

The results are expressed as mean ± SD, and Student's *t*-test was used for comparisons between two groups. Differences at different time points within groups were compared with repeated-measures ANOVA. Differences of survival rate in each group were analyzed by using Fisher exact probability. A two-tail value of *P* < 0.05 was considered significant. The experimental data were analyzed by SPSS 17.0 (SPSS Inc., Chicago, IL).

## 3. Result

### 3.1. Resuscitation Outcome and Survival Rates

**Figure 1:**
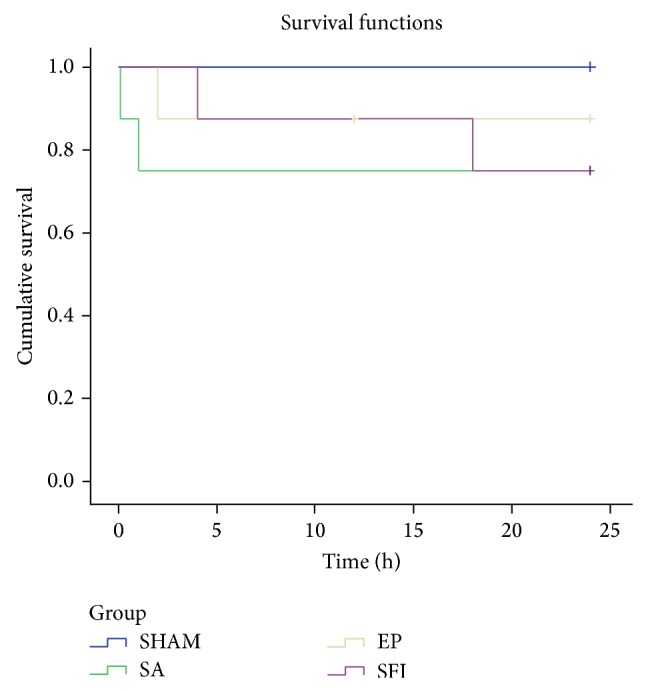
Cumulative survival in the sham and CPR (SA, EP, and SFI) groups. There were no significant differences in 6- and 24-hour survival rates between CPR groups.

Eighteen of 24 animals were successfully resuscitated in 3 CPR subgroups. Six animals in the SA group and 7 animals in the EP and SFI groups survived to 6 hours, and 6 animals in the 3 CPR subgroups survived to 24 hours. There were no significant differences in 6- and 24-hour survival rates between CPR groups. Survival curve in each animal group after ROSC is shown in Figure 1.

### 3.2. Hemodynamic Status

**Figure 2:**
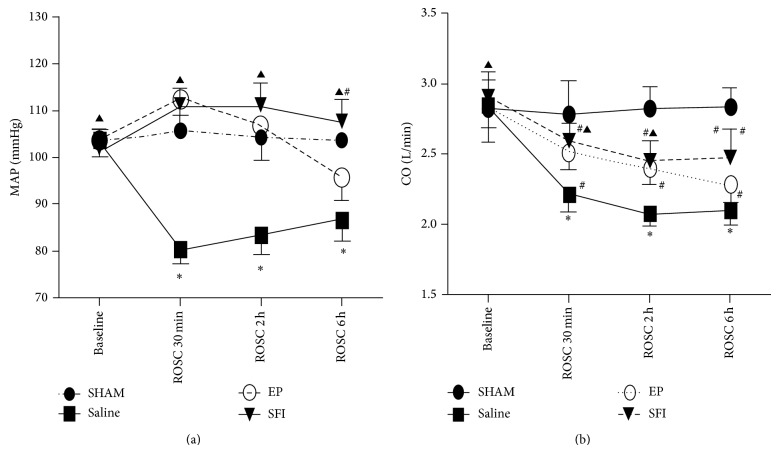
(a) Mean aortic pressure (MAP); (b) cardiac output (CO); the values are reported as mean (SD), ^*^ *P* < 0.05, and ^#^ *P* < 0.01 versus sham; ^▲^ *P* > 0.05 and ^#^ *P* < 0.01 versus EP (one-way repeated-measures ANOVA).

Baseline hemodynamics measurements are shown in Figure 2 among the four groups (*n* = 6 per group). MAP and CO did not differ significantly among four groups (*P* > 0.05). After successful resuscitation, the values of MAP were significantly decreased in the SA group between the baseline and 1, 2, or 6 h values (*P* < 0.01). In contrast, MAP was significantly increased in the SFI group compared to the EP group at 6 hours (*P* < 0.05) (Figure 2(a)). The values of CO were significantly decreased in the SA (*P* < 0.01) and EP and SFI (*P* < 0.05) groups after ROSC compared with the sham group. CO was significantly higher in the SFI and EP groups than the SA group at 30 min and 2 h (*P* < 0.05) (Figure 2(b)).

### 3.3. Effects of SFI on Mitochondrial Permeability Transition Pore (MPTP) and Mitochondrial Membrane Potential (MMP)

#### 3.3.1. Effects of SFI on Mitochondrial Membrane Potential (MMP)

**Figure 3:**
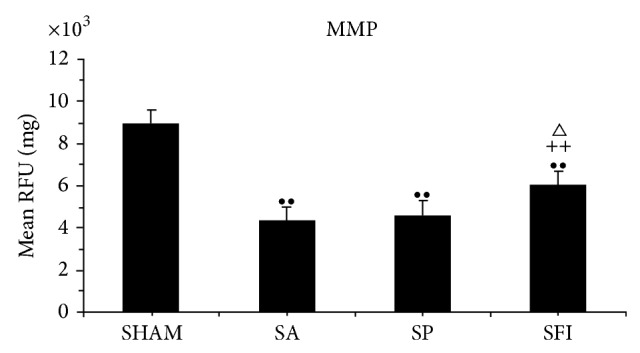
MMP are expressed as mean ± SEM (*n* = 6 in SHAM group and CPR groups). Quantitative data for MMP were significantly lower in the CPR subgroups than in the SHAM group (^••^ *P* < 0.01); MMP was significantly higher in the SFI group than in the SA group (^++^ *P* < 0.01); MMP was significantly higher in the SFI group than in the EP group (^△^ *P* < 0.05).

Compared with the SHAM group (Figure 3), cortical neurons in the cerebral frontal lobe showed significantly decreased level of MMP in all groups after 24 hours of CPR (*P* < 0.01). Compared with SA group and EP group, MMP in the SFI group elevated more significantly and there is no obvious statistical difference between the SA group and EP group.

#### 3.3.2. Effects of SFI on Mitochondrial Permeability Transition Pore (MPTP)

**Figure 4:**
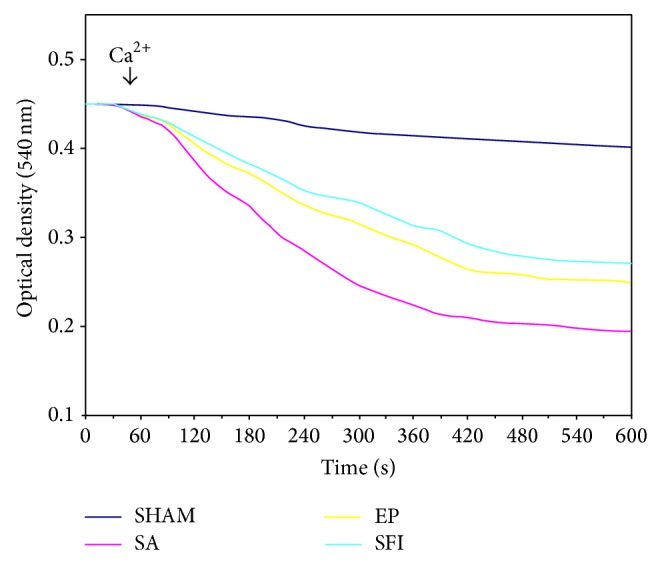
Changes of MPTP in pig cerebral cortex nerve cell. Mitochondria light density was significantly lower in all CPR groups than SHAM group at 24 hours after ROSC. In addition, the degree of opening of MPTP in SFI group is lighter than that in SA and EP group.

Compared with the SHAM group (Figure 4), cortical neurons in the cerebral frontal lobe showed significantly lower mitochondria light density in all groups after 24 hours of CPR, indicating obvious swelling of mitochondria and obvious opening of MPTP. In addition, the degree of opening of MPTP in SFI group is lighter than that in SA and EP group.

### 3.4. Neurological Deficit Scores (NDS) and Cerebral Performance Categories (CPC) Scores

The NDS and CPC score in the SFI group were all lower than that in the SA group and the EP group (Table 1), but there is no statistical difference (*P* > 0.05).

### 3.5. Effects of SFI on Mitochondrial Ultrastructure of Nerve Cells in Frontal Cortex

**Figure 5:**
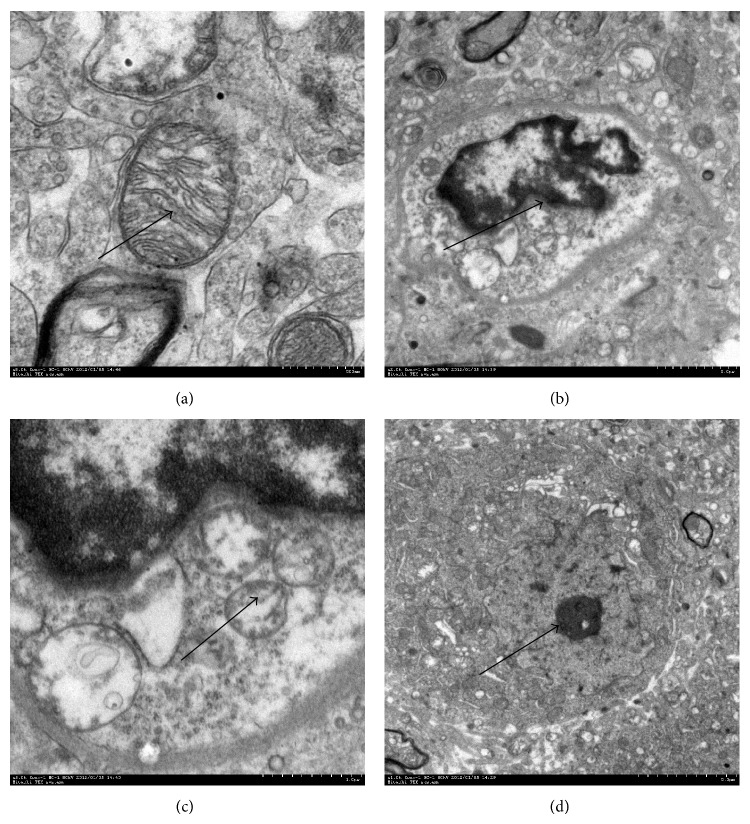
Cytoplasmic ultrastructure of the brain neuron under an electron microscope. (a) Normal cells structure of the brain neuron was observed in the SHAM group (arrows). (b) Ischemic pig brain cells ultrastructures in the SA group at 24 hours after cardiac resuscitation. Dotted arrows display brain glial cell nucleus damage, and the arrow shows mitochondrial swelling. (c) At 24 h after cardiac resuscitation in the EP group, mitochondrial architecture of brain neuron became more severe. (d) Mitochondrial architecture exhibited little intracellular damage in the SFI group at 24 hours after cardiac resuscitation.

Figure 5(a) (SHAM group) shows integrate mitochondrial structure and normal matrix.  Figure 5(b) (SA group) shows moderate impaired mitochondria manifested as mitochondrial crista fracture, matrix swelling and uneven density.  Figure 5(c) (EP group) shows severe impaired mitochondria manifested as mitochondrial crista fractured significantly and the electronic density decreased significantly.  Figure 5(d) (SFI group) shows slightly impaired mitochondria manifested as normal mitochondria, slightly swelling matrix and uneven density.

## 4. Discussion

Postcardiac arrest brain injury is a result of initial ischemic injury followed by reperfusion injury occurring within hours and days after ROSC [14]. Gong et al. [3] found that the significantly impaired mitochondrial respiratory chain can lead to oxidative phosphorylation disorders. This may be attributed to the following reasons. Cardiac arrest results in interruption of blood flow, followed by oxygen supply reduction in brain and systemic hypoxia. The sharp reduction of aerobic metabolism results in insufficiency of adenosine triphosphate (ATP) in spite of increased anaerobic metabolism. Moreover, oxidative stress as well as calcium overload leads to oxidative phosphorylation disorders and ATP reduction or even depletion; these all lead to changes of mitochondrial structure and function, affecting functions of cells and organs. In the present study, we found that the brain functions of pigs were impaired significantly, presented as increase of CPC and NDS scores, severe damage of ultrastructure of brain tissue, decrease of MMP, and intensified opening of MPTP. This may be another important mechanism of mitochondrial structure and function changes.

Shen-Fu injection is a typical form of SFI decoction for intravenous medication. The effects of SFI are based on aconitine properties, supplemented by ginsenoside, which can reduce local inflammation and brain edema after cerebral ischemia [15]. The protective mechanisms of SFI against cerebral ischemia/reperfusion injury include reduced excitatory amino acid toxicity, blockade of Ca^2+^ overload, and improved antioxidant capacity [16]. Zhang et al. showed that SFI could alleviate cerebral injury after CPR by promoting the expression of neuron-specific enolase [11]. In present study, we found that Shen-Fu injection could exert protective effect on mitochondria by elevating MMP and adjusting opening of MPTP, which could thus reduce brain cell mitochondria damage, alleviate brain cell edema, and protect brain function.

Mitochondria are not only main places to produce ATP, but also important energy conversion organelles in cells. Mitochondria can convert generated energy into the form of electrochemical potential and store it in endomembrane. This leads to asymmetric distribution of protons and other ions and thus forms MMP [17]. Normal MMP is a prerequisite for maintaining mitochondrial ATP production and the stability of MMP is conducive for cells to maintain normal physiological functions [18]. Previous research indicates that cytokines and mediators resulting in apoptosis are associated with decline of MMP. The process is mediated by cytochrome C on the mitochondrial membrane, followed by activation of caspase-3 to degrade DNA and cell apoptosis [19]. Other research proved that the decline of MMP is one of important indicators for cell apoptosis. The decline of MMP can cause the mitochondria respiratory chain to produce a large amount of active oxygen and thus lead to the irreversible process of apoptosis [20]. In the present study, we found that the MMP of neural cells of ROSC pigs declined significantly, indicating that the decline of mitochondrial MMP could be one of the main mechanisms of brain injury after ROSC. Nerve cells suffered severe ischemia-reperfusion injury after cardiac arrest and a large amount of inflammatory mediators and cytokines released. These resulted in decline of MMP, activation of caspase-3 apoptosis pathway, and mitochondria injury and apoptosis of brain cells. We found in our research that compared with SA group and EP group SFI could exert brain-protective effect by elevating MMP significantly. This may be explained that SFI could inhibit inflammatory responses, inhibit excessive release of cytokines, and inhibit activation of caspase-3 mediated apoptosis pathway. In our previous study, the multiple components of SFI could develop immune-modulatory effects; SFI could improve cell immune function by modulating the Th1/Th2 imbalance, reducing the expression of TNF-*α* to block the vicious circle of inflammatory response [14]. Moreover, SFI could inhibit Bcl-2, Bax, and caspase-3 expression and reduce cell apoptosis [12].

To date, the opening of MPTP is deemed as the common pathway for cell apoptosis and death after injury [21]. Stress conditions result in mitochondria injury and opening of MPTP, which can lead to increased permeability of mitochondrial membrane and mitochondrial swelling [22, 23]. The ion gradient disappears, the mitochondrial membrane potential collapses, the respiratory chain and oxidative phosphorylation uncouple, and then the ATP synthesis stops. Therefore, the opening of MPTP could be a key factor determining the destiny of injured neural cells after ischemia-reperfusion injury [24]. In the present study, we found that compared with SHAM group increased opening of MPTP could be detected in pig cerebral cortical neurons, indicating obvious swelling of mitochondria after ROSC, and these could be attributed to calcium overload, large amount of oxygen free radicals, and reduction of ATP [25]. Moreover, we found that in the SFI group the opening of MPTP and the swelling of mitochondria alleviated significantly, suggesting that SFI could affect the structure of MPTP and inhibit its opening. This could be an important protective mechanism of SFI against cerebral ischemia-reperfusion injury.

Epinephrine (EP) has been used for more than a century and is the recommended drug in the current guidelines for treatment of cardiac arrest. Epinephrine is a mixed adrenergic agonist, acting on *α*-adrenergic (*α*_1_ and *α*_2_) and *β*-adrenergic (*β*_1_ and *β*_2_) receptors, and is now being widely used in the treatment of cardiac arrest, allergic shock, and asthma [26]. The important mechanisms of EP for ROSC are mostly mediated by the *α*-adrenergic pathway, which increases coronary perfusion pressure, maintains peripheral vascular tension, and prevents arteriolar collapse, thus increasing the rate of ROSC. In contrast to the *α*-adrenergic receptor effects, *β*-adrenergic receptor stimulation has been suggested to have a deleterious effect: increasing oxygen consumption (VO_2_) of the myocardium, reducing subendocardial perfusion, and associating with postresuscitation myocardial dysfunction [10, 26]. Owing to these effects, increasing researches using EP have been unable to demonstrate a clinical benefit and have even suggested that this form of treatment may be harmful. Nowadays, researches in increasing numbers are dedicated to find alternative medicines for resuscitation. SFI, a traditional Chinese herbal medicine, has been commonly used in China for nearly 800 years. One of its main components, aconite, contains noradrenaline salsolinol, which not only has excitatory effects on p receptors and *α*-adrenergic receptors but also can increase coronary and cerebral blood flow, further effectively promote a reduction in blood pressure, and stabilize heart rate [10]. In the present study, the SFI group had better hemodynamic parameters compared with the EP and SA groups. Furthermore, in the present study, EP effectively reduced the time to ROSC compared with the SA group, although there was no effect on MMP, cerebral edema, or brain damage. By contrast, SFI injection suppressed the opening of MPTP and increased MMP, thus alleviating swelling of mitochondria and cells. Because of the multiple components of SFI, these effects may be mediated by, at least in part, improved ROSC and brain function.

## 5. Conclusions

Our research proved that there is mitochondria dysfunction of the pig brain cortex in the early stage of CPR. SFI treatment could not only alleviate postresuscitation mitochondria dysfunction caused by ischemia-reperfusion injury but also improve ultrastructure of nerve cells and alleviated postresuscitation brain cell damage.

## Figures and Tables

**Table 1:** 24-hour neurological outcome.

Outcome	SA	EP	SFI	*P*
CPC 1				
CPC 2	●	●●●	●●●●	
CPC 3	●●●	●●	●●	
CPC 4	●●	●		
CPC 5	●	●●	●●	
No ROSC	●			

Survival to 24 h	6	6	6	0.866^▲^
Good neurologic outcome	1	3	4	0.273^▲^
NDS	212.5 ± 88.0	148.3 ± 102.2	108.3 ± 74.9	0.160^▲^

NDS, neurological deficit scores: a score of 0 is defined as normal and a score of 400 is defined as brain dead; CPC, cerebral performance categories scores. The values represent mean ± SE, ^▲^ *P* > 0.05, versus SA or EP (Student's *t*-test).
